# Supplementary material for: Site specific target binding controls RNA cleavage efficiency by the Kaposi's sarcoma-associated herpesvirus endonuclease SOX
Source: Nucleic Acids Res. 2018 Oct 13;46(22):11968–79. doi: 10.1093/nar/gky932 (PMC6294519; doi:10.1093/nar/gky932)
Supplement: Supplementary Data [file gky932_supplemental_files.zip › clean_Supplementary Iegends FINAL 9.12.18_.docx]

**Supplementary Information**

**Figure S1.**

(**A**) Chromatograph of an S200 size exclusion run of KSHV SOX. SOX eluted predominately as a monomer as determined by molecular weight standards. Inset shows a Coomassie stained SDS-PAGE gel of the >95 pure SOX protein. (**B**) Chromatograph of an S200 size exclusion run of SOX mutant P176S. A similar elution profile is observed for P176S when compared to WT SOX. (**C**) Chromatograph of an S200 Size exclusion run of SOX mutant F179A. A similar elution profile is observed for F197A when compared to WT SOX. (**D**) Raw data used to generate the curve in Figure 1C. Urea-PAGE gel showing the effect of adding increasing concentrations of CaCl_2_ to reactions containing 2 μM of SOX in reaction buffer containing 0.7 mM MgCl_2_ and 5’-^32^P-labeled *LIMD1-54* RNA. Input lane indicates reaction conditions with 5’-^32^P *LIMD1-54* alone. (**E**) A time course using an active site mutant of SOX (D221N/E244Q) in the presence of 5’-^32^P *LIMD1-54* over the course of 1 h at low (2 μM) and high (10 μM) concentrations of SOX. Reactions were quenched every 6 minutes.

**Figure S2.**

In-line probing analysis of the *GFP*-100 RNA. The RNA was loaded directly (NR, no reaction), subjected to cleavage by RNase T1 or alkaline hydrolysis (-OH), or incubated at room temperature for 24 h or 48 h at pH 8.3 (in-line reaction, Rxn). Samples were separated on an 8 % urea PAGE gel. Accessible or unpaired regions show cleavage bands whereas structured regions stay blank (purple and green colored). The *GFP*-100 RNA structure deduced from the probing gel is shown in the lower right.

**Figure S3.**

(**A**) Domain architecture of SOX and locations of the host shutoff mutants. (**B**) 3D structure and position of SOX host shutoff mutants (PDB ID: 5HSW). Residues P176 and F179 are located within the bridge region of SOX close to the active site. Mutations P179S and F179A are thought to disrupt RNA binding; RNA substrate is hidden for clarity. Inset shows that residues P176 and F179 coordinate an adenine of the RNA substrate.

**Figure S4.**

Electrophoretic mobility shift assays (EMSA) of WT (**A**), P176S (**B**), or F179A (**C**) SOX using a 5’ ^32^P-labeled *LIMD1-54* RNA probe. Each reaction was run in triplicate using a 5% native PAGE gel. Tick marks labeled as a, b, and c indicate mobility shifts. Higher mobility shifts could indicate multimerization (ticks b,c) while lower shifts might indicate monomer binding (tick a). Gels were dried and quantified by taking the ratio of bound to unbound *LIMD1-54*.

**Figure S5.**

Kinetic characterization of SOX binding to WT *LIMD1-*54 **(A)**, the *LIMD1*-54 mutants *LIMD1 A-G cut site* (**B**), *LIMD1 3xA-G* (**C**), and *LIMD1 Zipper* (**D**), and the miRNA precursor *K2-31* (**E**). All RNAs were 3’ end labeled with biotin and conjugated to avidin-coated biosensors. Biosensors were then incubated in a well containing SOX to determine on-rates before being transferred to a well containing buffer only to determine off-rates. Red curves represent the statistical fitting of each curve, while the black and colored lines show experimental data.

**Figure S6.**

In line probing of the *LIMD1* Zipper and 3xA-G mutants to determine the secondary structures shown in Figure 5A. Lanes 1-5 represent the in-line probing analysis for the *LIMD1* Zipper mutant (AAA 🡪 ACA) and lanes 6-10 show the analysis for *LIMD1* 3xA-G. The RNA was loaded directly (NR, no reaction, lanes 1, 6), subjected to cleavage by RNase T1 (lanes 5, 10) or alkaline hydrolysis (-OH, lanes 3, 4, 8, 9), or incubated at room temperature for 24 hours (lanes 2, 7) at pH 8.3 (in-line reaction, Rxn). Samples were separated on an 8% urea PAGE analytical gel. Accessible or unpaired regions generate cleavage bands, whereas structured regions remain blank (orange and green colored). The loop region eliminated in the Zipper mutant is marked by green arrows.

**Figure S7**

Biochemical analysis of *LIMD1*-54 Zipper 2 mutant RNA. (**A**) The predicted folding of the *LIMD1*-54 zipper 2 RNA was determined using mFold (35). The residues involved in cut site recognition and the polyadenosine stretch are colored in red and orange, respectively. The red arrow marks the predicted SOX cleavage site and the black arrow indicates the position of the zipper 2 mutation, shown in green. (**B**) Binding kinetics of *LIMD1*-54 zipper 2 mutant. The table insert indicates protein concentration tested. Red curves represent the statistical fitting of each curve, while the blue and color lines show experimental data. **(C)** Catalytic efficiency of WT SOX was determined at a constant enzyme concentration (2 μM) using the 5’ ^32^P-labeled *LIMD1-54* Zipper 2 RNA probe. Experiments were performed in triplicate.

**Table S1.**

Nucleotide sequences of the RNA substrates used in the binding and turnover assays. Modifications made to either the 3’ or 5’ ends of the RNA are labeled accordingly.

**Table S2.**

Statistical analysis of the Bio-layer interferometry (BLI) data from Figures S4A-E. All samples were analyzed using BLI-Octet system software to determine raw binding kinetic data.
